# Temperature Dependence of the Indirect Gap and the Direct Optical Transitions at the High-Symmetry Point of the Brillouin Zone and Band Nesting in MoS_2_, MoSe_2_, MoTe_2_, WS_2_, and WSe_2_ Crystals

**DOI:** 10.1021/acs.jpcc.2c01044

**Published:** 2022-03-16

**Authors:** J. Kopaczek, S. Zelewski, K. Yumigeta, R. Sailus, S. Tongay, R. Kudrawiec

**Affiliations:** † Department of Semiconductor Materials Engineering, Faculty of Fundamental Problems of Technology, Wroclaw University of Science and Technology, Wybrzeże Stanisława Wyspiańskiego 27, 50-370 Wrocław, Poland; ‡ Materials Science and Engineering, School for Engineering of Matter, Transport and Energy, Arizona State University, Tempe, Arizona 85287, United States

## Abstract

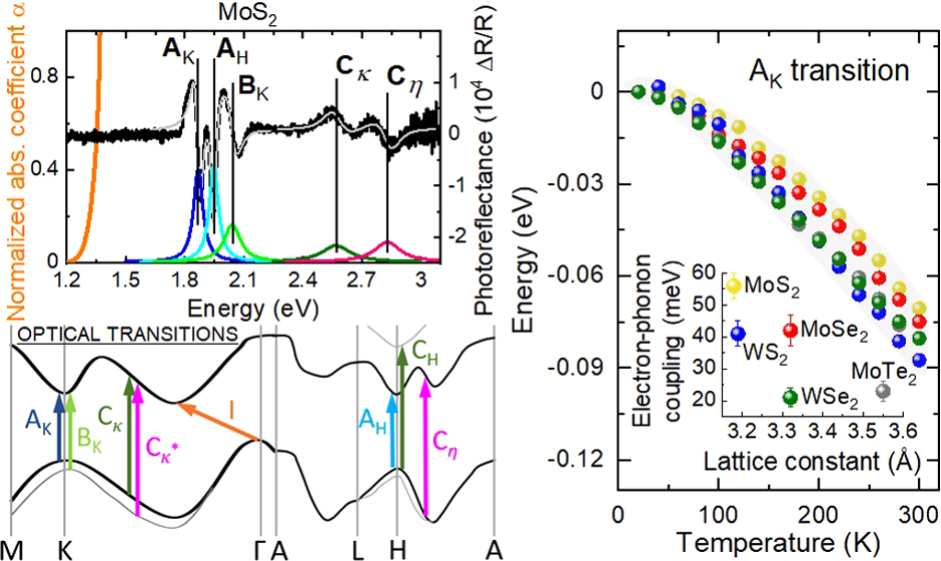

Following the rise of interest in the properties of transition metal dichalcogenides, many experimental techniques were employed to research them. However, the temperature dependencies of optical transitions, especially those related to band nesting, were not analyzed in detail for many of them. Here, we present successful studies utilizing the photoreflectance method, which, due to its derivative and absorption-like character, allows investigating direct optical transitions at the high-symmetry point of the Brillouin zone and band nesting. By studying the mentioned optical transitions with temperature from 20 to 300 K, we tracked changes in the electronic band structure for the common transition metal dichalcogenides (TMDs), namely, MoS_2_, MoSe_2_, MoTe_2_, WS_2_, and WSe_2_. Moreover, transmission and photoacoustic spectroscopies were also employed to investigate the indirect gap in these crystals. For all observed optical transitions assigned to specific *k*-points of the Brillouin zone, their temperature dependencies were analyzed using the Varshni relation and Bose–Einstein expression. It was shown that the temperature energy shift for the transition associated with band nesting is smaller when compared with the one at high-symmetry point, revealing reduced average electron–phonon interaction strength.

## Introduction

Among many semiconductor materials studied in recent years, atomically thin two-dimensional (2D) transition metal dichalcogenides (TMDs) attracted much attention owing to their unique properties in the quantum confinement limit such as the formation of room temperature excitons,^1^ world record exciton binding energies,^2,3^ spin-valley degree of freedom (valleytronics),^4^ and more recently, the formation of Moire excitons^5,6^ and also Bose–Einstein exciton–polariton condensates.^7^ While, as mentioned, these 2D TMDs offer unique research opportunities in their monolayer limit, including observation of optical transitions and their response to the interlayer coupling,^8−12^ there are only a few studies for TMD crystals in the bulk limit when adjacent layers are coupled to each other through vdW interactions.^13−16^ However, most of those works are based on piezoreflectance spectroscopy (i.e., another modulation technique, where strain in the crystals is periodically perturbated), which requires gluing studying materials to piezoceramics. Moreover, none of the mentioned articles present the evolution of indirect optical transition with temperature. From a fundamental perspective, studies of a variety of optical transitions (i.a. K → K, K → Γ, Γ → K−Γ, and at other van hove singularities^17,18^) for bulk TMDs crystals offer opportunities and bring challenges to understand the behavior of the electronic band structure. Here, the photoreflectance (PR) spectroscopy technique allows probing these hard to detect (by emission-like techniques) transitions by periodic modulation of electric field on the surface of 2D materials and subsequent detection of changes in the reflected light from that surface by a detector coupled to a lock-in amplifier.

In this work, we utilize temperature-resolved PR spectroscopy to access optical transitions between extended states across MX_2_ materials (where M = Mo, W and X = S, Se, Te) in the broad spectral range from the indirect to direct transitions, including band-nesting points. The mentioned band-nesting regions in the Brillouin zone (BZ) are related to out-of-high-symmetry points, where conduction and valence bands are parallel.^19,20^ Moreover, we employ absorption and photoacoustic (PA) measurements to study indirect transitions, where the latter is very sensitive to the indirect gap.^21^ To quantitatively describe measured temperature-related changes in the electronic band structure at the high-symmetry point of BZ and band nesting, we use Varshni and Bose–Einstein relations. By analyzing the obtained parameters for transitions of different nature, we have concluded about the strength of electron–phonon coupling and analyzed how sensitive optical transitions are to temperature changes. These studies extend the fundamental understanding of temperature effects on a variety of optical transitions in MX_2_ vdW layers.

## Experimental Methods

All samples studied here, which crystallize in the 2H phase, were grown at high temperatures (900–1100 °C) and low pressure (∼10^–6^ Torr) by vapor transport methods, with the assistance of iodine. The quartz ampoules (of 15 cm in length) during the growth process were subjected to inhomogeneous temperature, i.e., ∼50 °C difference between the hot and cold zones, which initiates the nucleation process and drives precursor transport. To remove any contamination from the growth ampoules, they were cleaned in piranha solution and annealed in H_2_ gas. The used precursors were mixed in a 1:2.05 M/X stoichiometric ratio, with the presence of iodine piece being a transport agent. The high quality of crystals and their 2H phase were confirmed by Raman and X-ray diffraction (XRD) measurements presented in the supplementary information (Figures S1 and S2).

Relative changes in the reflection coefficient (Δ*R*/*R*), namely the PR signal, were measured using the lock-in technique, which allows extracting: (i) weak AC signals proportional to Δ*R* from the background and (ii) the DC component (proportional to reflectance, *R*).^22,23^ Both components were detected by a Si PIN photodiode. The mentioned changes in the reflectance spectrum were evoked by modulation of the electric field on the surface of the investigated sample. For this purpose, the sample was illuminated by a laser beam (CW 405 nm line), which was mechanically chopped with a frequency of 285 Hz. All measurements were performed in the so-called “bright configuration,”^23^ where the sample was first illuminated by a spectrum of white light from a halogen lamp (150 W). Subsequently, the reflected white light was directed by a set of lenses onto a 0.55 m focal length monochromator entrance.

The absorption spectra were obtained based on transmission measurements in a so-called “dark configuration” experimental setup, i.e., a probe beam was first dispersed by a 0.3 m monochromator and later illuminated the sample. Afterward, the transmitted light was directed by lenses onto a Si PIN photodiode and measured using the lock-in technique. In Section II of the Supporting Information, we have provided schemes presenting both “dark and bright configuration” on the example of transmission measurements.

The PA spectroscopy measurements were carried out on the same experimental setup where transmission investigations were performed. However, in this case, after illuminating a sample with the periodically modulated monochromatic light, the pressure oscillations of the surrounding gas were detected by an acoustic transducer (electret microphone) and measured with the lock-in method. The mentioned pressure oscillations were induced due to heat transfer generated from nonradiative processes from the sample to the surrounding gas.^21^

To obtain temperature dependencies from 20 to 300 K of PR and transmission spectra, samples were mounted on a cold finger inside a cryostat working in a helium closed-cycle refrigerator coupled with a programmable temperature controller.

## Results and Discussion

**Figure 1:**
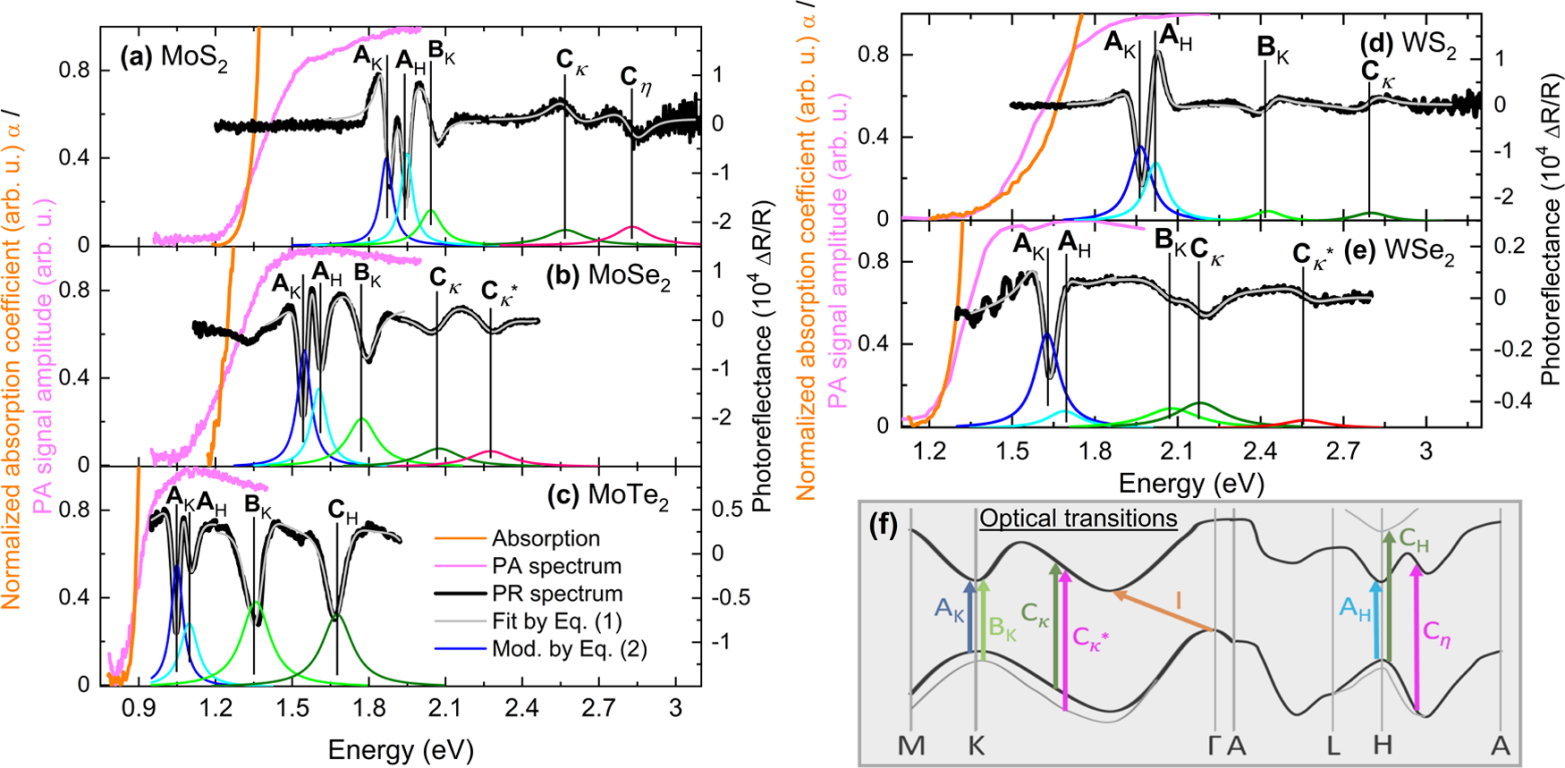
Experimental PR (black line), PA (magenta line), and absorption (orange line) spectra obtained at room temperature for (a) MoS_2_, (b) MoSe_2_, (c) MoTe_2_, (d) WS_2_, and (e) WSe_2_. The PR spectra were fitted by Aspnes expression eq 1 (gray line). For a better illustration, the moduli of transitions (colored lines) were depicted on the bottom of every panel. (f) Scheme of optical transitions observed in the experiment.

The primary aim of this work is to study the evolution of the electronic band structure with temperature by means of tracking energy variation obtained for optical features related to the different points of BZ. For that purpose, it is essential to assign unambiguously optical transitions to given *k*-points of BZ, which was done based on the results of our previous studies. In those articles, we have identified different features by comparing their pressure coefficient obtained experimentally from PR measurements with hydrostatic pressure and theoretically within density functional theory (DFT).^24,25^ In Figure 1, we show PR spectra (black line) obtained at room temperature, together with the results of the fitting procedure by eq 1 (gray line). Furthermore, the PA and absorption spectra (magenta and orange lines, respectively) are presented in this figure.

It can be seen that the absorption edge related to the fundamental indirect gap red-shifts following chemical trends as the lattice constants increase when transition metal or chalcogen atom is substituted with a heavier element.^21^ Similar behavior is visible for direct transitions related to: (i) H and K high-symmetry points of the BZ, namely, the A_H_, A_K_, and B_K_ transitions, where the latter arises due to spin–orbit splitting of the valence band edge^26,27^ and (ii) band nesting, i.e., C_K_ transition. The mentioned H high-symmetry point may only be observed for the bulk samples since it does not exist in the BZ of the monolayers crystals.^28^ The A_H_ transition can overlap with the spectral features related to the excited state of the A_K_ exciton or the interlayer exciton, which are expected to occur at similar energy.^28−30^ However, the temperature evolution of the PR spectrum discussed later shows that the intensity of this spectral feature is not well correlated with the intensity of the PR resonance attributed to the A_K_ transition. Hence, this resonance cannot be related to the excited state of the A_K_ exciton or interlayer exciton and therefore is assigned to the A_H_ transition.^28^ Moreover, It is worth noticing that B_K_ transition, which arises due to spin–orbit splitting, should also be present. However, since the energy difference between B_K_ and B_H_ is small (up to 15 meV^31^) and broadening large (∼80 meV), it is not possible to distinguish the B_H_ feature. The contribution to the features labeled as C can come from a different area of BZ: (i) the band nesting at the path between K → Γ transition C_K_ (for MoS_2_, MoSe_2_, WS_2_, and WSe_2_), where C_K_* is from a lower valence band, (ii) the band nesting at the path between H → A transition Cη (for MoS_2_),^32^ or (iii) H high-symmetry point (for MoTe_2_) transition C_H_ to a higher conduction band.^25^ Although we have assigned the origin of the C transition for each crystal, the mentioned contribution can be more complicated and may come from a different area of BZ simultaneously for one feature. A more detailed analysis of this energy range of PR spectra is not possible due to the significant broadening (∼100 meV) of mentioned transitions. To distinguish between features related to the high-symmetry point of the BZ and band nesting, we have used Latin or Greek letters, respectively, as the subscript of the transition label.

The parameters, such as energy or broadening, describing optical transitions observed in the PR spectra (Figure 1), were determined by fitting the relative changes of the reflection coefficient by eq 1, i.e., the Aspnes formula^33^
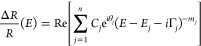
1where the shape of the resonance (optical feature) is represented by amplitude (*C*), phase (θ), and broadening (Γ), whereas *E* is its energy position, the parameter (*m*) in the above formula is related to the type of optical transition, and for the excitonic one is equal to 2. Additionally, on the bottom part of each panel in Figure 1, we have depicted the moduli for direct transitions obtained by eq 2 with parameters determined based on the fitting procedure by the Aspnes formula.
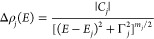
2Since the area under the curve representing moduli depends not only on the oscillatory strength for a given transition but also on how sensitive this transition is to the modulation of the built-in electric field, the quantitative comparison of the strength of different features is rather difficult. Nevertheless, it can be noticed that the broadening of transitions corresponding to the band nesting is higher than that for the one related to high-symmetry points of the BZ. This situation stems from the contribution to the former transition from the extended *k*-space region.^32^ The value of broadening parameter (Γ) for the features associated with the high-symmetry points of the BZ is about 30–60 meV and increases with temperature by 20–40 meV, whereas for the transitions related to band nesting, the initial broadening is ∼60–100 meV and increases by 30–45 meV in the studied temperature range.

**Figure 2:**
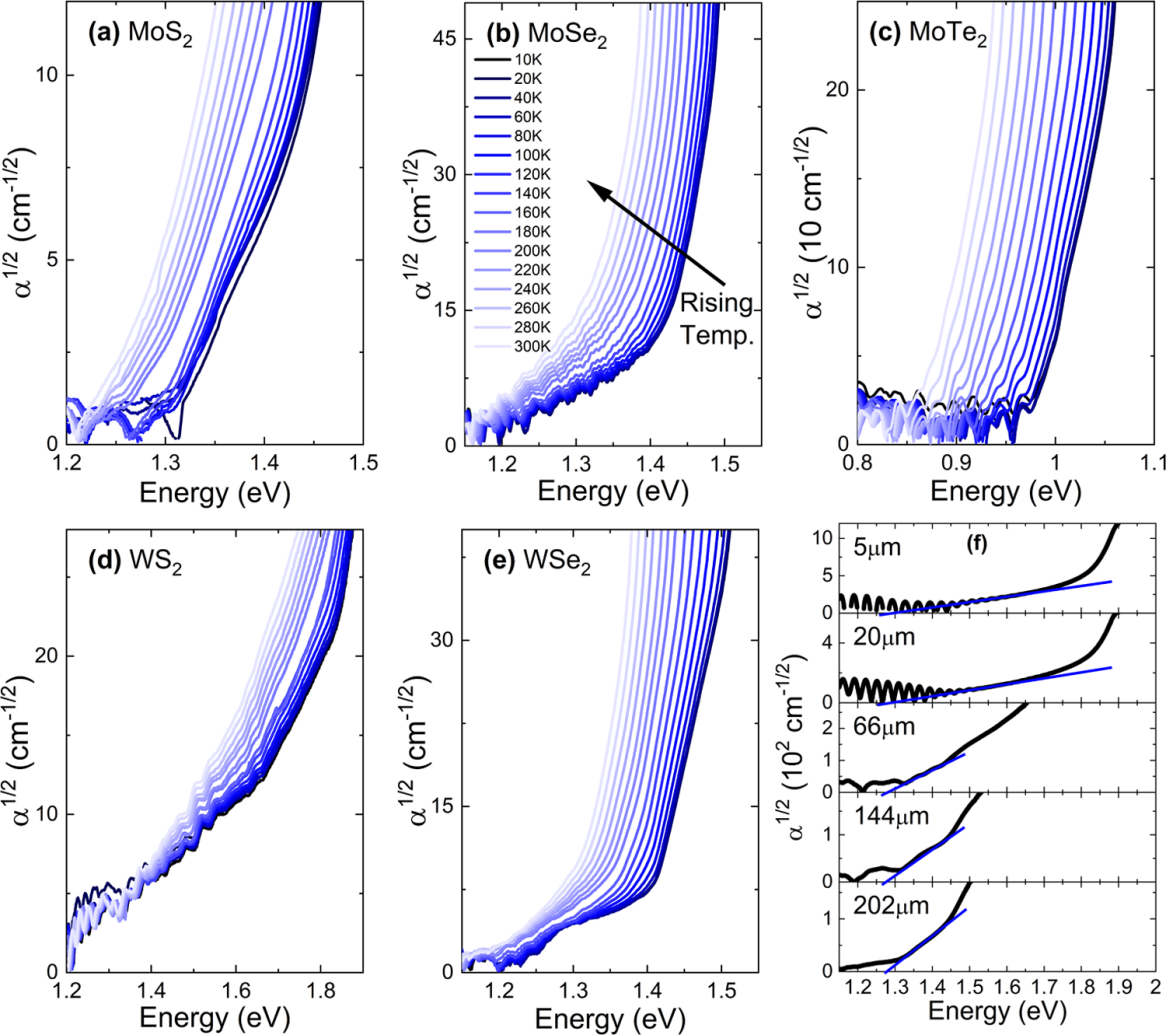
Temperature dependencies of absorption spectra for crystals studied in this work (a–e) and (f) the thickness dependence of absorption spectra obtained for MoS_2_.

For studies of the indirect gap with temperature performed based on the transmission measurements, samples of proper thickness must have been prepared. Such an approach was required since, for a thin sample (<50 μm), the signal corresponding to indirect absorption was too weak to be measurable, and primarily the direct absorption edge was visible, as shown in Figure 2f.

Moreover, for thinner samples (5 and 20 μm), the Fabry–Perot oscillations were presented, complicating even more determination of the position of the indirect absorption edge. To avoid the issues mentioned above and consider the differences in the extinction coefficient in the vicinity of absorption edge for each material, samples of thickness around 150–200 μm were prepared. Temperature dependencies obtained with the temperature absorption spectra for all investigated crystals are presented in Figure 2a–e. To determine the energy of the indirect gap, we have used a linear extrapolation (blue line in Figure 2f) of the absorption edge associated with the emission of the phonon since the second process, namely corresponding to its absorption, was too weak to be observed.

**Figure 3:**
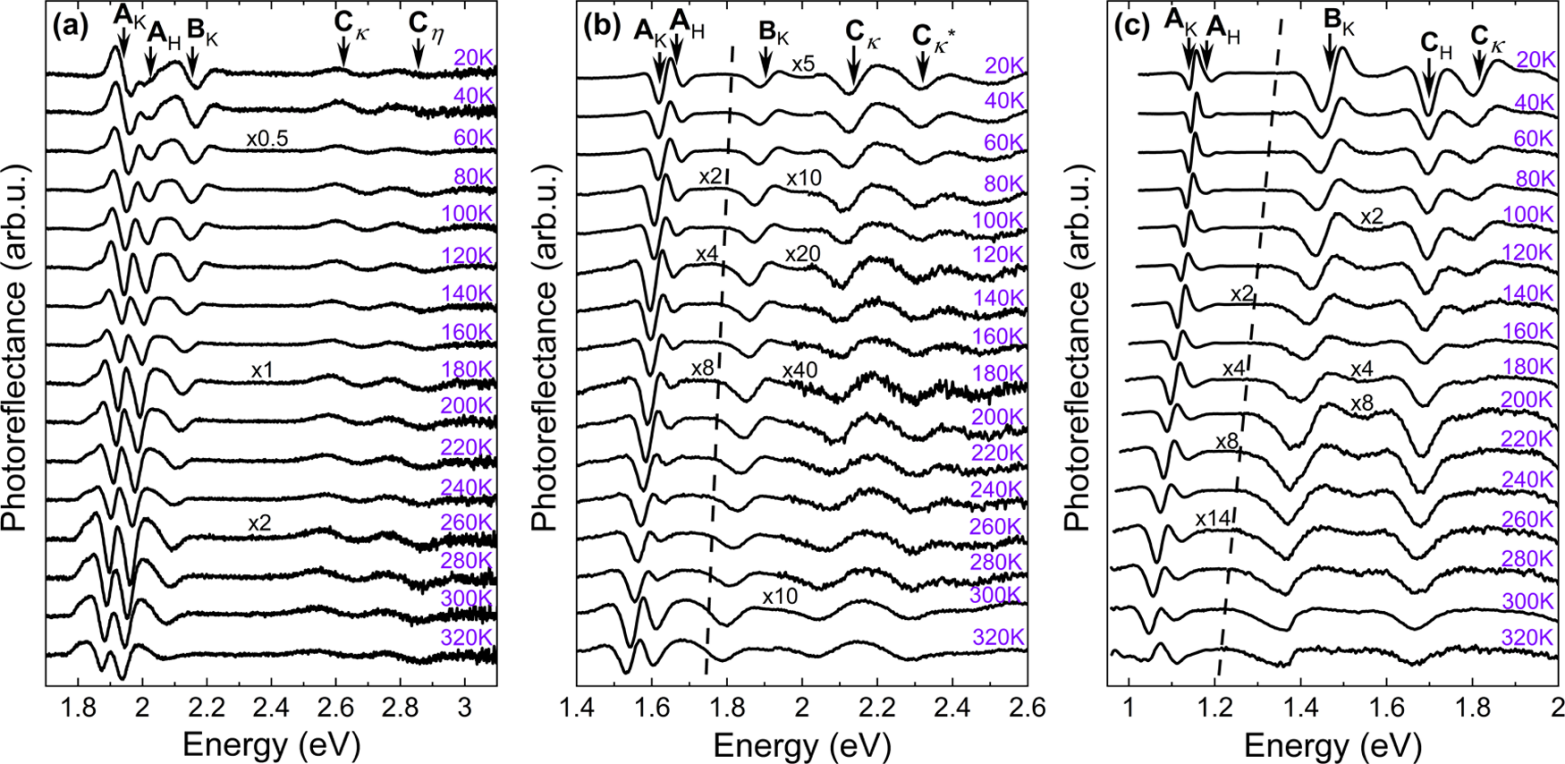
PR spectra of (a) MoS_2_, (b) MoSe_2_, and (c) MoTe_2_ measured with temperature from 20 to 300 K. On top of every dependency, the assignment of optical transitions is presented, with arrows indicating the energy position of each transition obtained from the fitting procedure by eq 1.

**Figure 4:**
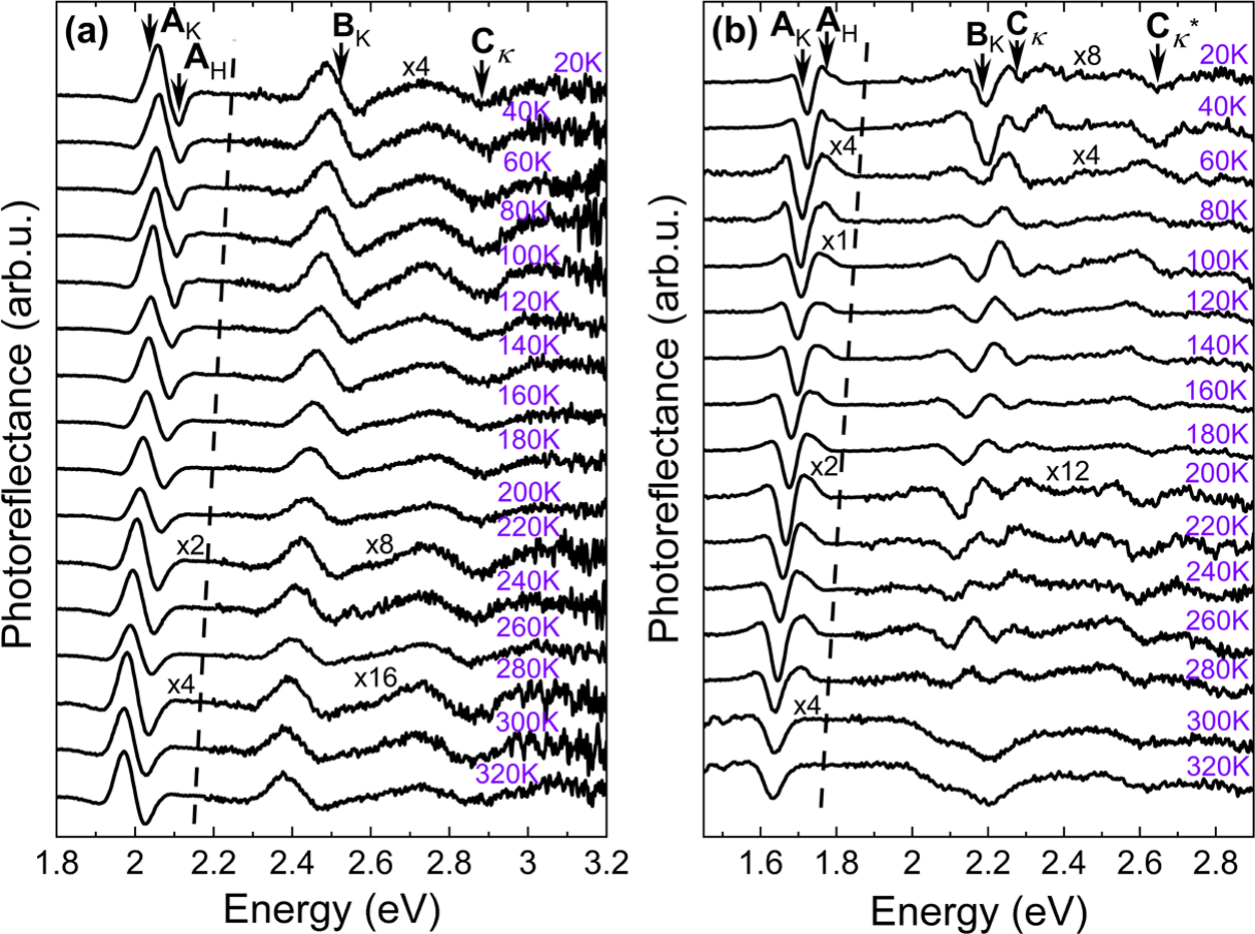
Temperature dependency of PR spectra of (a) WS_2_ and (b) WSe_2_ measured with temperature from 20 to 300 K. On top of every dependency, the assignment of optical transitions is presented, with arrows indicating the energy position of each transition obtained from the fitting procedure by eq 1.

The PR spectra were also measured from 20 to 300 K to determine temperature dependencies of the direct optical transitions; the results of experiments are plotted in Figure 3 (MoS_2_, MoSe_2_, and MoTe_2_) and Figure 4 (WSe_2_ and WSe_2_).

**Figure 5:**
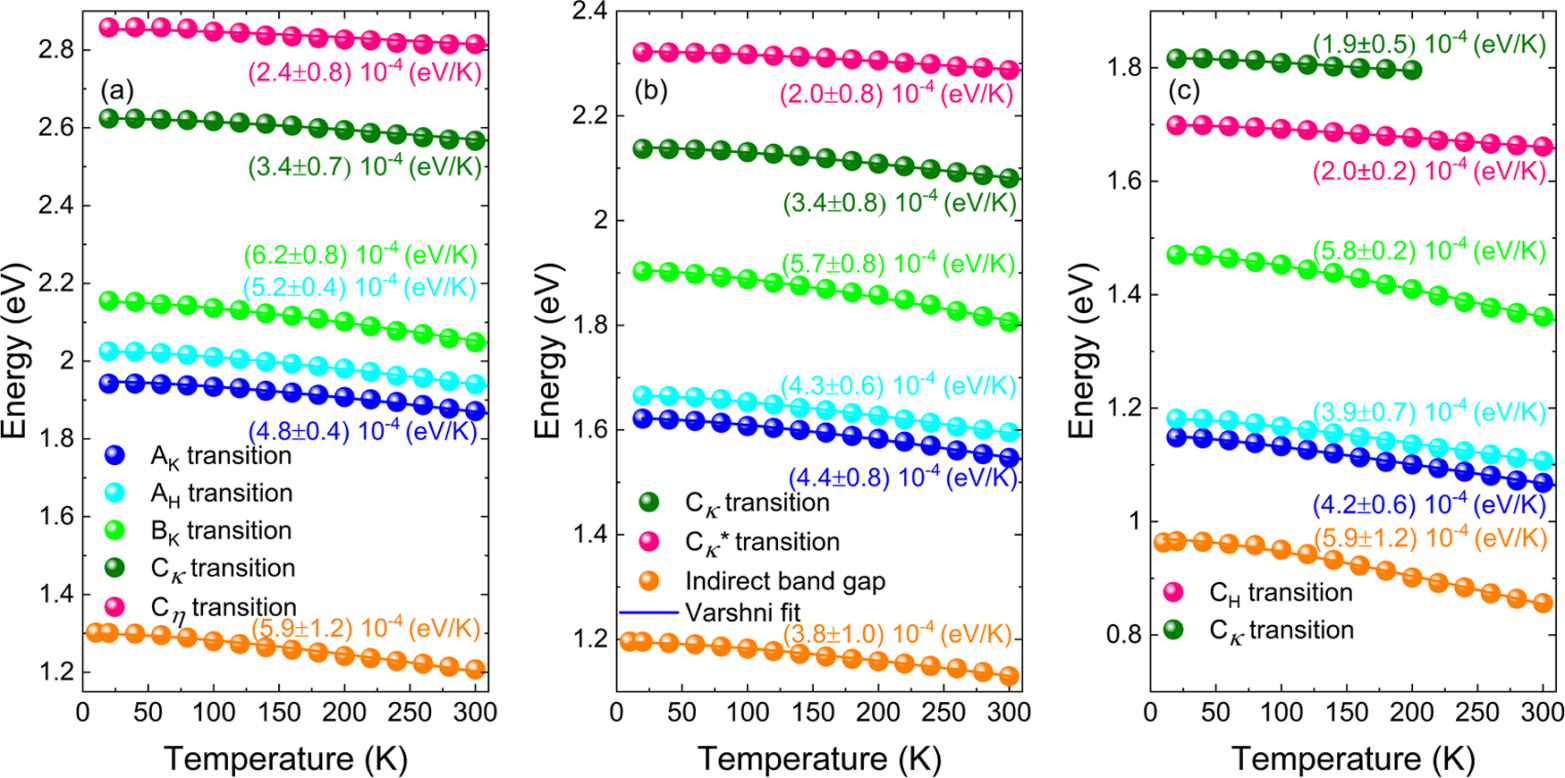
Temperature dependencies of energy of (a) MoS_2_, (b) MoSe_2_, and (c) MoTe_2_, extracted with the fitting procedure, for all observed optical features. For clarity, only the Varshni relation was depicted for every dependency along with the α temperature coefficient. The error bars are within the size of experimental points.

**Figure 6:**
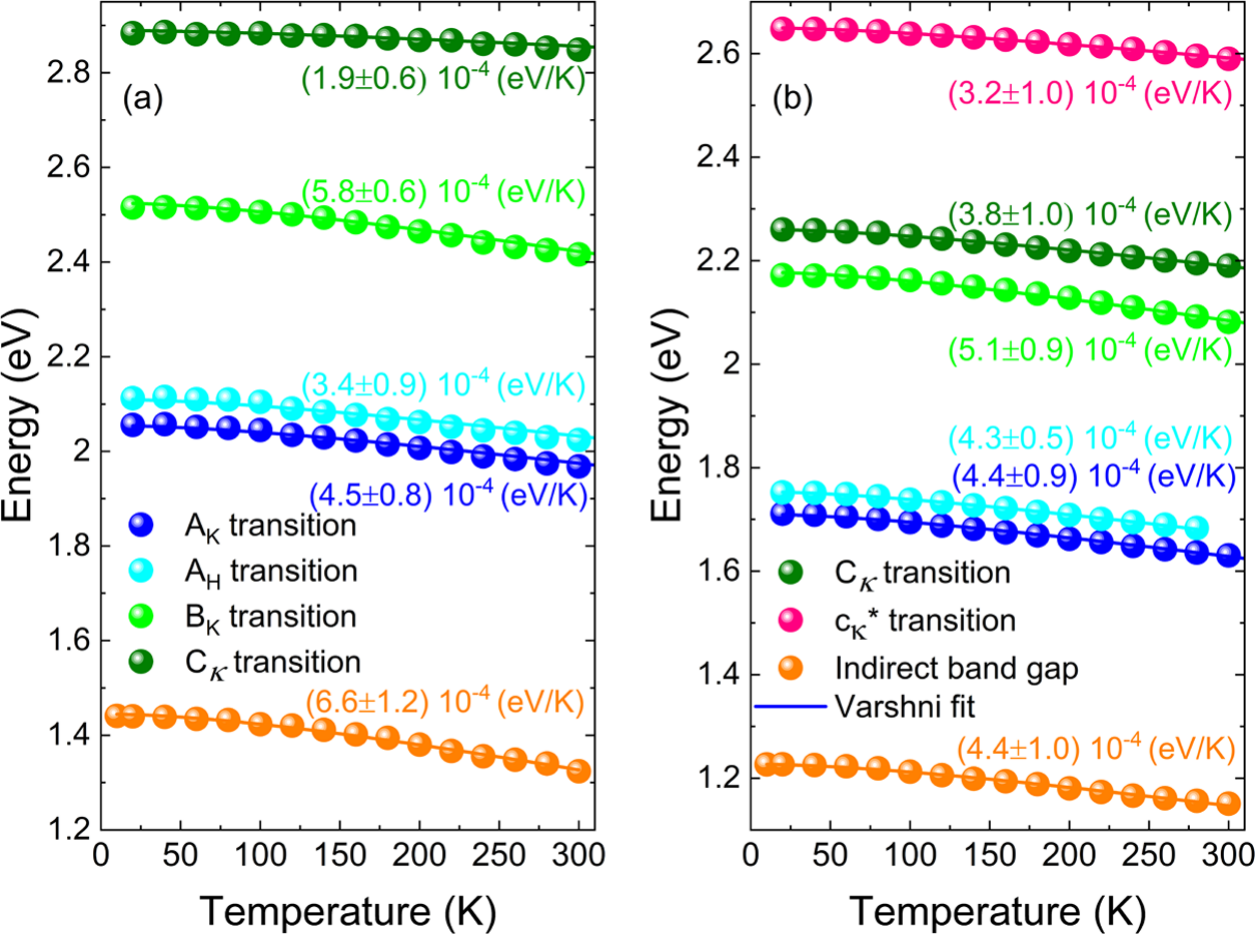
Evolution of energy with temperature obtained for (a) WS_2_ and (b) WSe_2_ and extracted from the fitting procedure for all observed optical features. For clarity, only the Varshni relation was depicted for every dependency together with the α temperature coefficient. The error bars are within the size of experimental points.

Observed optical features red-shift with increasing temperature for all investigated crystals, however, with a different rate when comparing transition corresponding to the high-symmetry point and band nesting. Additionally, it can be seen that the amplitude of these features decreases as they broaden due to increased electron–phonon interaction. For the purpose of a detailed description of the observed behavior of direct transitions with temperature, we have first applied a fitting procedure using eq 1 for each spectrum. The determined dependencies presented in Figures 5 and 6 were subsequently fitted by Varshni empirical relation (solid lines), eq 3, and Bose–Einstein formula, eq 4, to quantitatively analyze temperature evolution of optical features
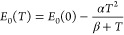
3
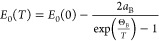
4where *E*_0_(0) is the extrapolated value of the transition energy at 0 K in both Equations. The α and β fitting parameters are so-called Varshni empirical coefficients in the formula 3, whereas considering the Bose–Einstein relation 4, the *a*_B_ and Θ_B_ are the strength of the electron-average phonon interaction and the average phonon temperature.

In Table 1, we have summarized the determined parameters from the fitting procedure using eqs 3 and 4. The obtained parameters for A and B exciton overlap, including their uncertainties, with previously reported values.^34^ Considering the temperature behavior of transition involving out-of-high-symmetry points of BZ, our studies provide such results for the first time.

**Table 1:** Varshni and Bose–Einstein Parameters Extracted for Transitions of Different Nature from the Fitting Procedure by Equations 3 and 4 for All Studied Materials^d^

sample	transition	*E*_0_ (eV)	α (10^–4^ eV/K)	β (K)	*E*_0_ (eV)	*a*_B_ (meV)	Θ_B_ (K)
MoS_2_	I	1.299 ± 0.020	5.9 ± 1.2	fixed 250	1.297 ± 0.020	44 ± 16	200 ± 90
	A_K_	1.944 ± 0.001	4.8 ± 0.8	250 ± 80	1.945 ± 0.002	56 ± 4	240 ± 40
	A_H_	2.026 ± 0.002	5.2 ± 0.4	fixed 250	2.023 ± 0.001	49 ± 6	230 ± 20
	B_K_	2.154 ± 0.003	6.2 ± 0.8	fixed 250	2.153 ± 0.001	52 ± 6	210 ± 20
	Cκ	2.625 ± 0.002	3.4 ± 0.7	fixed 250	2.624 ± 0.001	24 ± 4	190 ± 40
	Cη	2.854 ± 0.011	2.4 ± 0.8	fixed 250	2.854 ± 0.009	16 ± 6	180 ± 80

sample	transition	*E*_0_ (eV)	α (10^–4^ eV/K)	β (K)	*E*_0_ (eV)	*a*_B_ (meV)	Θ_B_ (K)
MoSe_2_	I	1.194 ± 0.020	3.8 ± 1.0	fixed 230	1.194 ± 0.020	25 ± 10	180 ± 80
	A_K_	1.623 ± 0.002	4.4 ± 0.8	230 ± 60	1.620 ± 0.001	42 ± 5	210 ± 30
	A_H_	1.665 ± 0.003	4.3 ± 0.6	fixed 230	1.665 ± 0.001	31 ± 5	190 ± 20
	B_K_	1.906 ± 0.003	5.7 ± 0.8	fixed 230	1.901 ± 0.002	57 ± 10	240 ± 50
	Cκ	2.139 ± 0.002	3.4 ± 0.8	fixed 230	2.139 ± 0.003	25 ± 10	190 ± 60
	Cκ*	2.323 ± 0.005	2.0 ± 0.8	fixed 230	2.322 ± 0.002	17 ± 10	210 ± 60

sample	transition	*E*_0_ (eV)	α (10^–4^ eV/K)	β (K)	*E*_0_ (eV)	*a*_B_ (meV)	Θ_B_ (K)
MoTe_2_	I	0.969 ± 0.020	5.9 ± 1.2	fixed 160	0.966 ± 0.020	49 ± 12	190 ± 80
	A_K_	1.149 ± 0.002	4.2 ± 0.6	160 ± 80	1.147 ± 0.002	23 ± 3	160 ± 70
	A_H_	1.182 ± 0.004	3.9 ± 0.7	fixed 160	1.181 ± 0.002	26 ± 8	160 ± 40
	B_K_	1.474 ± 0.001	5.8 ± 0.2	fixed 160	1.470 ± 0.001	48 ± 4	190 ± 20
	C_H_	1.700 ± 0.001	2.0 ± 0.2	fixed 160	1.700 ± 0.001	13 ± 6	150 ± 30
	Cκ	1.817 ± 0.001	1.9 ± 0.5	fixed 160	1.816 ± 0.001	12 ± 4	150 ± 40

sample	transition	*E_0_* (eV)	α (10^–4^ eV/K)	β (K)	*E*_0_ (eV)	*a*_B_ (meV)	Θ_B_ (K)
WS_2_	I	1.445 ± 0.020	6.6 ± 1.2	fixed 210	1.440 ± 0.020	68 ± 18	240 ± 80
	A_K_	2.054 ± 0.008	4.5 ± 0.8	210 ± 80	2.055±0.003	41 ± 4	180 ± 50
	A_H_	2.110 ± 0.010	3.4 ± 0.9	fixed 210	2.111 ± 0.002	32 ± 10	170 ± 40
	B_K_	2.525 ± 0.003	5.8 ± 0.6	fixed 210	2.524 ± 0.001	46 ± 6	190 ± 30
	Cκ	2.850 ± 0.010	1.9 ± 0.6	fixed 210	2.850 ± 0.008	14 ± 6	180 ± 60

sample	transition	*E*_0_ (eV)	α (10^–4^ eV/K)	β (K)	*E*_0_ (eV)	*a*_B_ (meV)	Θ_B_ (K)
WSe_2_	I	1.227 ± 0.020	4.4 ± 1.0	fixed 190	1.227 ± 0.020	25 ± 12	150 ± 80
	A_K_	1.710 ± 0.004	4.4 ± 0.9	190 ± 80	1.710 ± 0.001	21 ± 3	150 ± 40
	A_H_	1.753 ± 0.002	4.3 ± 0.5	fixed 190	1.753 ± 0.001	25 ± 4	150 ± 20
	B_K_	2.178 ± 0.004	5.1 ± 0.9	fixed 190	2.174 ± 0.003	48 ± 12	220 ± 70
	Cκ	2.260 ± 0.003	3.8 ± 1.0	fixed 190	2.260 ± 0.003	22 ± 8	150 ± 60
	Cκ*	2.611 ± 0.005	3.2 ± 1.0	fixed 190	2.611 ± 0.005	20 ± 9	160 ± 60

sample	transition	*E*_0_ (eV)	α (10^–4^ eV/K)	β (K)	*E*_0_ (eV)	*a*_B_ (meV)	Θ_B_ (K)
MoS_2_^a^	A	1.935 ± 0.005	4.8 ± 0.5	180 ± 50	1.976 ± 0.020	46 ± 15	220 ± 50
	B	2.142 ± 0.005	4.7 ± 0.5	160 ± 50	2.179 ± 0.020	42 ± 8	200±30
WS_2_^a^	A	2.067 ± 0.005	4.7 ± 0.5	210 ± 50	2.102 ± 0.020	37 ± 10	200 ± 40
	B	2.493 ± 0.005	5.5 ± 0.6	200 ± 50	2.534 ± 0.020	45 ± 11	200 ± 35
GaAs^b^	*E*_g_	1.517	5.5	225	1.571	57	240
GaSb^b^	*E*_g_	0.813	3.78	94	0.811	22	127
InAs^b^	*E*_g_	0.419	2.8	93			
Si^c^	*E*_g_	1.170	4.73	636			

a Ref (34).

b Ref (35).

c Ref (36).

d Literature data are also provided for comparison.

Here (Table 1), we present an evident quantitative difference in the rate (the α parameter) at which transitions related to the high-symmetry point of BZ (A and B) and band nesting (Cκ and Cη) shift with temperature. Such behavior was also confirmed by the Bose–Einstein analysis (the *a*_B_ values) since the strength of electron-average phonon interaction is clearly reduced for the band-nesting transitions. The chemical trend for the electron–phonon coupling, i.e., the parameter *a*_B_, is quite challenging to analyze due to its large uncertainty. However, for the A_K_ transition, which is determined with the greatest relative accuracy, it can be stated that this coupling decreases with changes from lighter to heavier atoms, i.e., going from S to Se(Te) and from Mo to W.

In general, the magnitude of electron–phonon coupling may be defined as the extent to which distortion of the nuclei, along a vibrational coordinate, changes the energy separation between two electronic states.^37−40^ In our case, the electronic states are the valence and conduction bands, which are different in terms of symmetry of the wave functions for different optical transitions.^41,42^ Therefore, for the same crystal with the same phonon dispersion, the electron–phonon coupling may change for different optical transitions. In our case, such a situation is observed for all five crystals. In addition, a general trend is observed that the electron–phonon coupling for the band nesting-related transition is smaller than for the remaining transitions. When comparing the electron–phonon coupling for the same optical transition between different crystals, we mainly deal with different phonon dispersion.^43−45^ At the same time, the symmetry of the wave functions plays a minor role in these changes since it does not vary much, as shown from density functional theory calculations.^45,46^ As the mass of atoms increases (from S to Te), the phonon dispersion changes and the electron–phonon coupling change. Therefore, the observed electron–phonon coupling is weaker for compounds containing heavier atoms (MoS_2_ vs MoSe_2_ vs MoTe_2_ and WS_2_ vs WSe_2_). However, an accurate comparison of the electron–phonon coupling between different optical transitions and different materials requires a computation that takes into account all of the discussed aspects in a quantitative manner. Such calculations are not the subject of this article, but they may be interesting and may explain the observed changes in the *a*_B_ parameter. In our research, the main factor contributing to the evolution of the band gap with temperature is the variation in the crystal lattice constant. The electron–phonon coupling is an additional factor influencing the mentioned changes of the band gap. Therefore, it is worth mentioning that other experimental methods are more recommended for studying electron–phonon coupling.^47−49^

**Figure 7:**
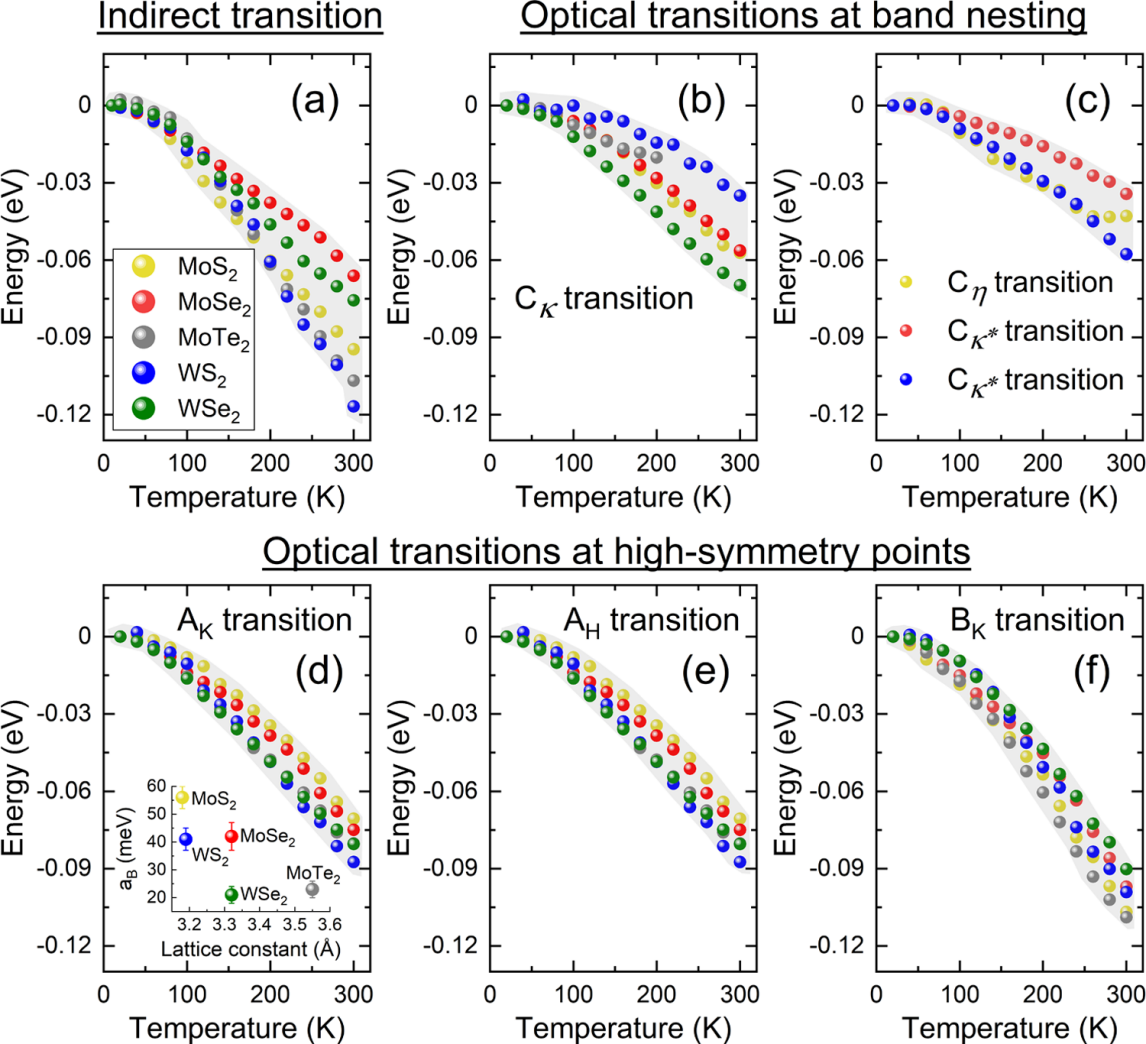
Temperature-related shift of (a) indirect transition, (b) C_K_, (c) C_K_* (observed for MoS_2_), Cη (observed for MoSe_2_ and WS_2_), (d) A_K_, (e) A_H_, and (f) B_K_ transition. The inset graph of panel (d) shows the dependency of the strength of the electron-average phonon interaction versus lattice constants taken from ref (50).

The average phonon temperatures (Θ_B_) are comparable for each transition as well as for each crystal, taking into account the considerable uncertainty with which this parameter was determined. Moreover, when comparing the range of obtained values of phonon temperature (Θ_B_) for vdWs crystal with data for binary III–V semiconductors (GaAs, GaSb), it can be seen that they are similar. Additionally, the strength of electron–phonon interaction, determined here for A_K_, A_H_, and B_K_ features, are close to the *a*_B_ value for GaSb and much lower than for GaAs; the same tendency is observed for α parameters. Moreover, values of both α and *a*_B_ parameters for transition corresponding to band nesting are even lower than for GaSb. That similarity in trends observed for α and *a*_B_ parameters are expected since the reduced electron–phonon interaction strength should induce smaller sensitivity of a given transition to temperature, and subsequently, the α temperature rate should also decrease. The described behavior is clearly visible in Figure 7, where the temperature-related energy shift of each transition is compared for different vdWs crystals.

As stated above, the chemical trends can be recognized for the A_K_ transition, i.e., for the crystal with a larger lattice constant, the α temperature coefficient, and the electron-average phonon interaction decrease (see inset graph of Figure 7d). Moreover, it can be seen that the transition associated with the out-of-high-symmetry point of the BZ zone (band nesting) shift weakly, in the studied temperature range, i.e., by ∼30–70 meV (Figure 7b,c), whereas the A_K_ and A_H_ red-shift by around 75–90 meV (Figure 7d,e). The most sensitive to temperature is the indirect and B transitions, the energy of which decreases by ∼90–110 meV Figure 7a,f. These observations are clear evidence for the reduced electron–phonon coupling for optical transitions related to band-nesting points in the BZ.

## Conclusions

In this work, we have presented studies on the evolution of the electronic band structure with temperature (from 20 to 300 K) of the most commonly investigated molybdenum- and tungsten-based TMDs: MoS_2_, MoSe_2_, MoTe_2_, WS_2_, and WSe_2_. The temperature-evoked changes at high-symmetry points of the BZ and band nestings were tracked by observing changes in the energy of optical transitions. For the mentioned purpose, we have utilized the PR technique for studying the direct optical transitions, whereas the PA spectroscopy and absorption measurements were used to analyze the indirect gap. The observed direct transitions were assigned to K/H points of the BZ and band nestings located at the path between K → Γ and H → A points. Moreover, the temperature dependencies of energy for indirect and direct optical transitions were determined following the performed experiments. We analyzed them in terms of Varshni and Bose–Einstein expressions for a quantitative understanding of the observed behavior. Based on the obtained parameters, we have concluded that the band-nesting transitions are less sensitive to changes of temperature for all vdW crystals studied here. This finding is related to the reduced strength of electron-average phonon interaction for this type of transition. Furthermore, the performed investigation shows that the PR technique can be successfully utilized to study TMD crystals with temperature, owing to the ability to probe above-gap optical transitions, leading to a better understanding of the behavior of this type of material.

## Supporting Information Available

Raman and XRD spectra of studied crystals scheme of “bright and dark configuration” of the experimental setup (PDF)
