# Supplementary material for: Temperature Dependence of the Indirect Gap and the Direct Optical Transitions at the High-Symmetry Point of the Brillouin Zone and Band Nesting in MoS2, MoSe2, MoTe2, WS2, and WSe2 Crystals
Source: J Phys Chem C Nanomater Interfaces. 2022 Mar 16;126(12):5665–74. doi: 10.1021/acs.jpcc.2c01044 (PMC8978178; doi:10.1021/acs.jpcc.2c01044)
Supplement: Supplementary file 1 — jp2c01044_si_001.pdf [file jp2c01044_si_001.pdf]

Supporting Information for Publication:

Temperature Dependence of the Indirect Gap and the Direct Optical Transitions at the High-symmetry Point of the Brillouin Zone and Band Nesting in MoS_2, $MoSe_2$, $MoTe_2$, WS_2, and WSe_2 Crystals

J. Kopaczek[1,2 a)], S. Zelewski[1], K. Yumigeta[2], R. Sailus[2], S. Tongay[2], and R. Kudrawiec[1]
[1]Department of Semiconductor Materials Engineering, Faculty of Fundamental Problems of Technology, Wroclaw University of Science and Technology, Wybrzeże Wyspiańskiego 27, 50-370 Wrocław, Poland
[2]Materials Science and Engineering, School for Engineering of Matter, Transport and Energy, Arizona State University, Tempe, Arizona 85287, USA
[a)] Corresponding author: jan.kopaczek@pwr.edu.pl

I. Raman and XRD results

The Raman spectra were measured in the backscattering configuration by a Renishaw inVia Raman Microscope (50x magnification), where CW 466nm laser line was used as an excitation beam. Obtained position of peaks for each crystal is in good agreement with the literature data confirming the 2H phase of studied materials. [1–5]

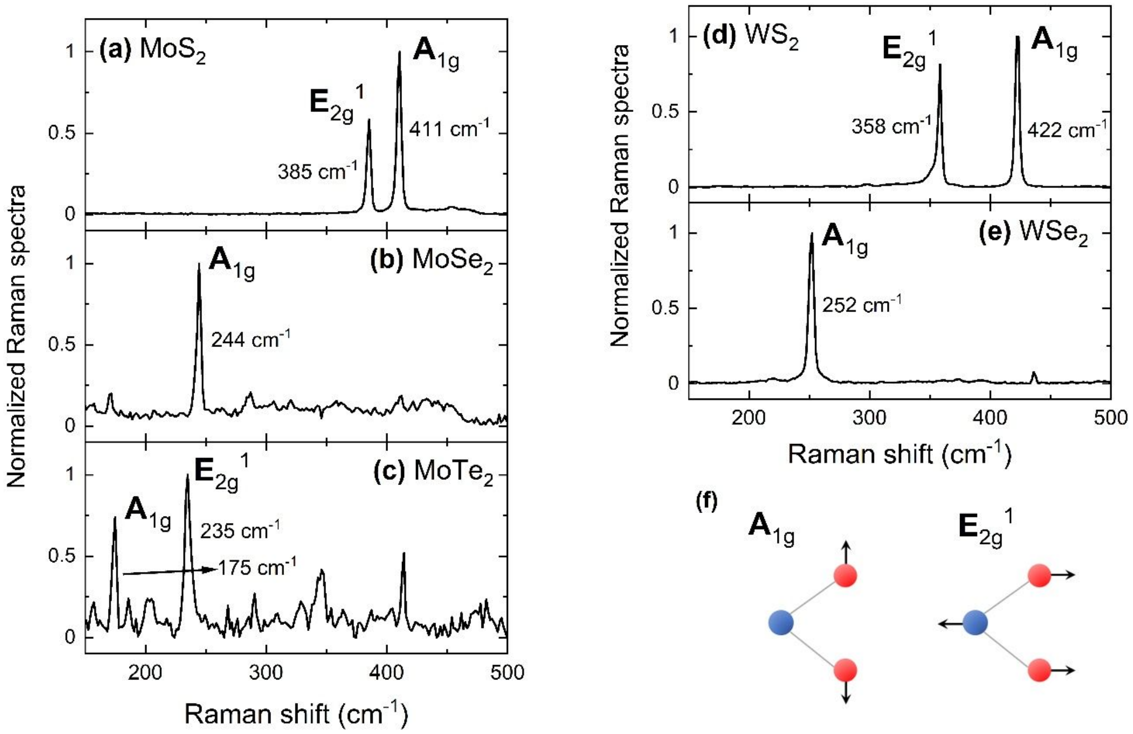

Figure S1. Raman spectra of (a) MoS_2, (b) $MoSe_2$, (c) $MoTe_2$, (d) WS_2, and (e) WSe_2 crystals obtained at room temperature. (f) Scheme of atoms vibrations for measured A_{1g} and E_{2g}^1 Raman modes.

Moreover, we have performed x-ray diffraction (XRD) measurements using Malvern PANalytical Aeris with Cu Kα radiation to characterize the crystal structure. The XRD spectra in Figure S2 show well-defined reflections from a set of (0,0,l) planes stacked along the c-axis lattice direction.

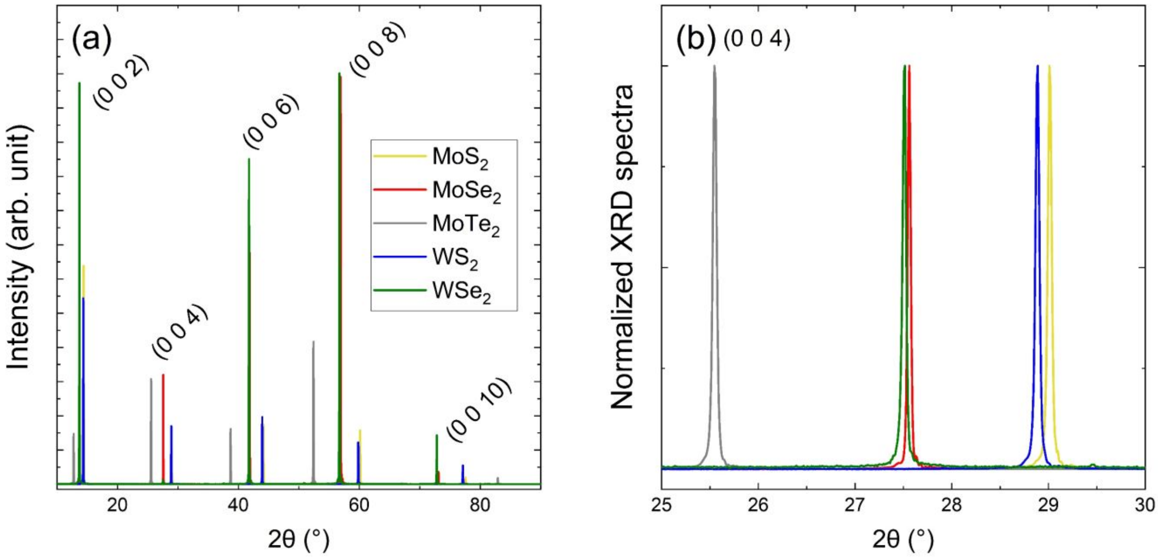

Figure S2. (a) The XRD spectra of studied in this works crystals, the low broadening of observed reflections confirms their high crystallinity. (b) The detailed XRD spectra of (004) plane reflections.

II. Methods

Here we present schemes of both "dark and bright" experimental configuration on the example of transmission measurements. In the "bright configuration" Figure S3, a probe beam emitted by a quartz tungsten halogen (QTH) lamp first interacts with a studied sample and then is directed to a monochromator. Subsequently, dispersed light is measured by a detector in a lock-in technique. In this configuration, a whole spectrum emitted by a QTH lamp illuminates the surface of a sample, which due to the photovoltaic effect, can cause a reduction of the built-in electric field. Since this effect is not relevant for studied crystals, a "bright configuration" was adopted in our work for PR measurements. Moreover, the detector is mounted directly onto the exit slit of the monochromator, which suppresses any background signals.

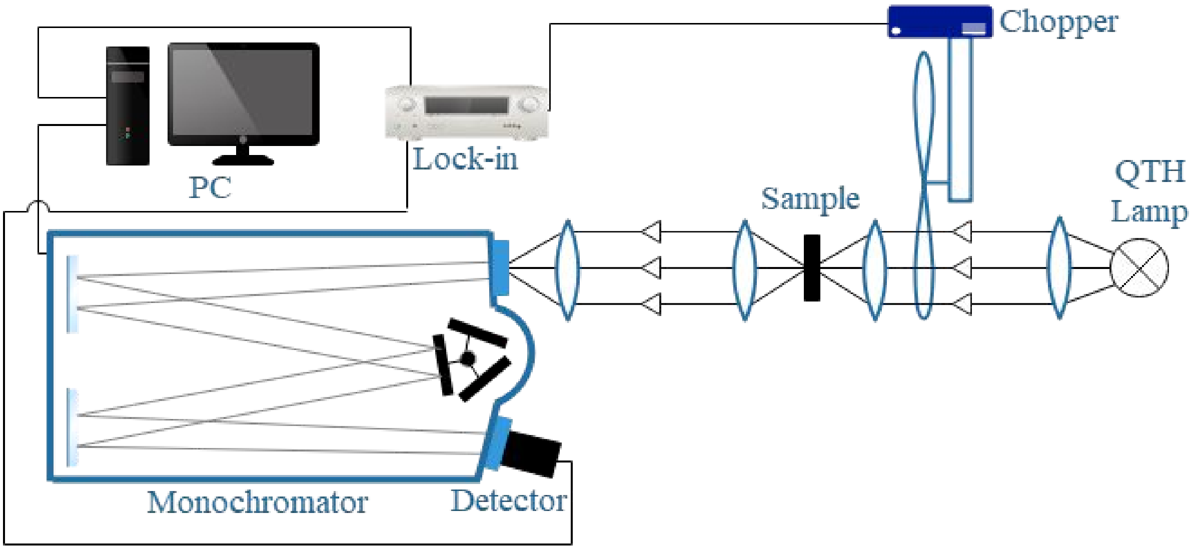

Figure S3. Experimental setup for transmission measurements in a "bright configuration".

In the “dark configuration” Figure S4, the probe beam emitted by the QTH lamp is first dispersed by a monochromator and then interacts with a sample. After reflection from the sample, the probe beam is directed by a set of lenses on a detector. With this approach, the background signal is not suppressed. Additionally, in the PR technique, a studied sample can emit light after laser excitation, leading to an occurrence of the constant background signal. On the other hand, when PR spectra are measured in a “bright configuration”, the emitted signal will be presented only at a specific spectral position. In the case of transmission measurements, we have used a “dark” configuration since the same experimental setup was used for the photoacoustic (PA) study. For that study, the “dark” configuration is required since we need to illuminate a sample by a monochromatic light to detect by electret microphone sound generated due to absorption of that light.

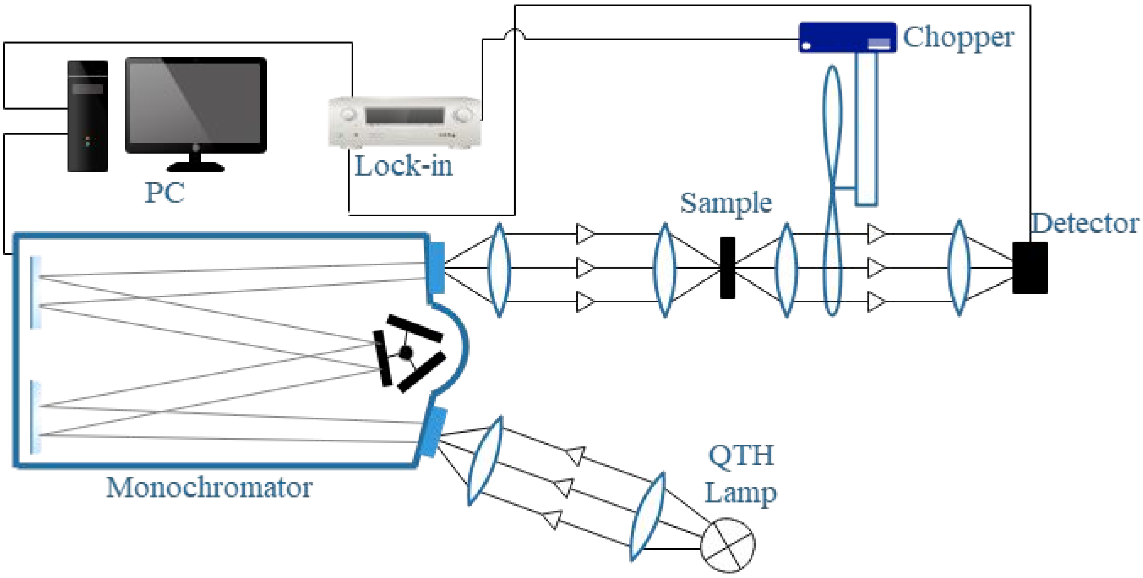

Figure S4. Experimental setup for transmission measurements in a “dark configuration”.

References:

(1) Zhao, W.; Ghorannevis, Z.; Amara, K. K.; Pang, J. R.; Toh, M.; Zhang, X.; Kloc, C.; Tan, P. H.; Eda, G. Lattice Dynamics in Mono- and Few-Layer Sheets of WS_2 and WSe_2. *Nanoscale* **2013**, *5* (20), 9677–9683.

(2) Zeng, H.; Zhu, B.; Liu, K.; Fan, J.; Cui, X.; Zhang, Q. M. Low-Frequency Raman Modes and Electronic Excitations in Atomically Thin MoS_2 Films. *Phys. Rev. B* **2012**, *86* (24), 241301.

(3) Terrones, H.; Corro, E. D.; Feng, S.; Poumirol, J. M.; Rhodes, D.; Smirnov, D.; Pradhan, N. R.; Lin, Z.; Nguyen, M. a. T.; Elías, A. L.; et al. New First Order Raman-Active Modes in Few Layered Transition Metal Dichalcogenides. *Sci. Rep.* **2014**, *4* (1), 4215.

(4) Ruppert, C.; Aslan, O. B.; Heinz, T. F. Optical Properties and Band Gap of Single- and Few-Layer $MoTe_2$ Crystals. *Nano Lett.* **2014**, *14* (11), 6231–6236.

(5) Tonndorf, P.; Schmidt, R.; Böttger, P.; Zhang, X.; Börner, J.; Liebig, A.; Albrecht, M.; Kloc, C.; Gordan, O.; Zahn, D. R. T.; et al. Photoluminescence Emission and Raman

Response of Monolayer MoS_2, $MoSe_2$, and WSe_2. *Opt. Express, OE* **2013**, *21* (4), 4908–4916.
